# Principle-Guided Psychotherapy for Children and Adolescents (FIRST): study protocol for a randomized controlled effectiveness trial in outpatient clinics

**DOI:** 10.1186/s13063-023-07717-y

**Published:** 2023-10-21

**Authors:** Abby Bailin, Evelyn Cho, Ariel Sternberg, Spencer C. Evans, Nathan L. Hollinsaid, Sarah Kate Bearman, John R. Weisz

**Affiliations:** 1 Department of Educational Psychology, The University of Texas at Austin, 1912 Speedway, Suite 5.708, Austin, TX 78712-1289 USA; 2 Department of Psychology, Harvard University, 33 Kirkland Street, Cambridge, MA 02318 USA; 3 University of Miami, 5665 Ponce de Leon Blvd, Coral Gables, FL 33146 USA

**Keywords:** Randomized controlled effectiveness trial, Children and adolescents, Implementation, Depression, Anxiety, Conduct problems, Trauma, Empirically supported treatment, Psychotherapy

## Abstract

**Background:**

Hundreds of youth psychotherapy randomized trials have generated scores of helpful empirically supported treatments (ESTs). However, the standardized structure of many ESTs and their focus on a single disorder or homogeneous cluster of problems may not be ideal for clinically referred youths who have comorbidity and whose treatment needs may shift from week to week. This concern has prompted development of flexible transdiagnostic, modular youth psychotherapies. One of these, designed for efficient training and implementation, is FIRST—a transdiagnostic intervention built on five empirically supported principles of change (i.e., feeling calm, increasing motivation, repairing thoughts, solving problems, and trying the opposite) and targeting common internalizing and externalizing youth mental health disorders and problems. FIRST has shown promise in improving youth mental health in three open trials. Now, in a more rigorous test, we seek to (1) conduct a randomized controlled trial comparing FIRST to usual care in real-world clinical practice settings; (2) examine a promising candidate mediator of change—regulation of negative emotions; and (3) explore variables that may influence clinicians’ treatment implementation.

**Methods:**

This is an assessor-naïve randomized controlled effectiveness trial in youth outpatient community clinics in New England and Texas. Using double randomization, clinic-employed clinicians and treatment-referred youths (7–15 years old) are independently randomly allocated (1:1) to FIRST or usual care. We aim to recruit 212 youth participants, all referred through normal community pathways, with elevated symptoms of anxiety, depression, conduct problems, or post-traumatic stress. This study will test the effectiveness of FIRST compared to usual care on mental health outcomes, examine whether those outcomes are mediated by regulation of negative emotions, and explore clinician factors that may be associated with FIRST implementation and outcomes. Session recordings are coded to assess treatment fidelity.

**Discussion:**

This study will evaluate the effectiveness of FIRST in youth community mental health settings, relative to the care usually provided in those settings. If FIRST is found to be effective, it could offer an efficient and practical method to increase use of empirically supported treatment principles in real-world practice contexts.

**Trial registration:**

NIH Clinical Trials Registry, NCT04725721. Registered 27 January 2021, https://clinicaltrials.gov/ct2/show/study/NCT04725721

**Supplementary Information:**

The online version contains supplementary material available at 10.1186/s13063-023-07717-y.

## Principle-Guided Psychotherapy for Children and Adolescents (FIRST): study protocol for a randomized controlled effectiveness trial in outpatient clinics

Research across five decades has produced more than 500 published randomized controlled trials (RCTs) of youth psychotherapies—accompanied by scores of manual-guided protocols—many of which have been shown to effectively improve youths’ clinical outcomes and functioning and are thus deemed empirically supported treatments (ESTs). A meta-analysis of youth ESTs spanning more than three decades found a mean post-treatment effect size of 0.726 [1], indicating a medium-to-large effect [2]. However, when ESTs are tested in real world clinical practice settings against usual care (UC; i.e., everyday practices commonly employed in clinical settings) the overall effect size is much smaller (0.295) [3, 4]. This steep drop-off in effectiveness when ESTs are moved from laboratory to real world—termed the implementation cliff [5]—as well as evidence of limited uptake by community providers [6] suggests that some ESTs may need adjustments in design, delivery, and implementation to boost their effects in typical practice contexts. Many ESTs are lengthy, linear (i.e., using a relatively standardized sequence of sessions), and target only a single disorder or problem (or a cluster of related problems). While these approaches are efficacious and highly useful in many ways (e.g., treatment fidelity, replication of research), they pose at least five challenges to effective implementation in everyday clinical care.

First, clinically referred youths often present with multiple problems or comorbidities, yet most ESTs for youths focus on a single disorder or a small family of related problems [7]. Additionally, clinician caseloads span a wide range of problem areas, making learning one EST protocol—let alone several—highly inefficient [8]. Second, training clinicians to deliver one EST for one disorder can take several days, and therefore many separate trainings (which would be required for training in multiple EST protocols) would be needed to address most clinicians’ complex caseloads [9]. Third, most youth ESTs have a standardized linear design with a prescribed series of skills delivered in a relatively fixed order of sessions. This progression may not work well in everyday practice, where youths’ most pressing problems may change from week to week, requiring treatments that flexibly shift to address such fluctuations. Fourth, the typical recommended number of sessions for youth ESTs exceeds the average number attended by youth treated in community settings. Although the average EST includes 16 sessions spanning 16 weeks [1], most youths in outpatient care attend fewer than 8 sessions [10–12]. Accordingly, there is a need for more efficient treatments that maximize therapeutic benefits before attendance wanes. Fifth, although youth ESTs have been shown to effectively reduce youths’ mental health symptoms, little is known about the mechanisms underlying these effects [13]. Identifying such mechanisms could lead to a sea change in our understanding of how ESTs work, and what the active ingredients are, informing steps to make treatments more efficient and potentially more effective.

### Innovation

Efforts to address these barriers have prompted innovative approaches to youth psychotherapy treatment design, delivery, and implementation to match the real-world practice contexts. This has included the development of multi-diagnostic, modular treatments, such as the *Modular Approach to Therapy for Children with Anxiety, Depression, Trauma, and Conduct* (MATCH) [14]. MATCH offers clinicians a menu of 33 modules comprising core procedures from effective ESTs for various youth mental health concerns. Modules are grouped within the diagnostic categories for which they are used (e.g., exposure for anxiety, problem solving for depression). MATCH has shown significant effects relative to outpatient UC in two randomized controlled effectiveness trials (RCETs) [15, 16]. Other studies, however, have highlighted concerns about the lengthy (5–6 days) and costly training required, and findings have suggested that MATCH may be difficult to deliver effectively without a high level of implementation support (e.g., individual consultation) that is likely unsustainable in everyday clinical practice [17–19].

In response to these implementation challenges, we sought to develop a more efficient treatment for youths by drawing on empirically supported principles of change (ESPCs) [20–25] that can be applied across a range of disorders and problems. Our approach, *Principle-Guided Psychotherapy for Children and Adolescents: The FIRST Program* (hereafter FIRST) is a principle-guided, transdiagnostic treatment for youths [26], developed to address the aforementioned barriers to the delivery of ESTs in routine practice settings. The potential value of leveraging ESPCs has been articulated by members of the Society of Clinical Psychology EST Task Force [25] and others [e.g., 20, 21, 23, 24]. In short, although learning specific treatment techniques and procedures may be useful for clinicians, knowing *why* they are using these techniques (e.g., what kind of change process is needed to produce symptom reduction) may prove even more beneficial. Knowledge of ESPCs may be particularly important for community-based clinicians for whom EST approaches may be new [20–25, 27], or may represent a divergence from their usual clinical practice [23]. This rationale undergirds FIRST, which is based on five ESPCs supported by decades of research [28].

### Description of FIRST

The five core elements of FIRST are as follows:

1. Feeling Calm (calming, relaxation, emotion regulation),
2. Increasing Motivation (incentivizing behavior change, e.g., via attention, praise, or rewards),
3. Repairing Thoughts (cognitive reappraisal),
4. Solving Problems (systematic steps of problem solving), and
5. Trying the Opposite (practicing positive opposites that lead to corrective experiences, e.g., behavioral activation for depression, exposure for anxious avoidance).

Each principle can be flexibly and efficiently applied to the treatment of problems spanning depression, anxiety, obsessive–compulsive disorder, post-traumatic stress, and conduct problems among youth ages 7 to 15. FIRST was designed with input from experienced community clinicians and feedback from independent treatment developers and researchers [29].

FIRST has been tested in three open trials using low-cost training and clinician support. In the initial trial, Weisz and colleagues [29] tested FIRST in two community outpatient clinics with 24 community-referred youths. Treated youths had an average of 2.21 K-SADS diagnoses before receiving FIRST. At the end of treatment, 81% no longer met criteria for their primary diagnosis. Two subsequent open trials were conducted by Cho and colleagues [8] in an outpatient university training clinic with treatment duration limited to six sessions. In the first of these trials, FIRST was delivered to 22 youths ages 7 to 17. In the second, FIRST was delivered to 26 youths ages 11 to 17. In both trials, youths demonstrated improvements on youth- and caregiver-reported functional top problems and clinical symptom severity from session-to-session, as well as from pre-to-post on measures of both internalizing and externalizing symptoms. In all three trials, FIRST showed evidence of acceptability and feasibility [8, 29].

Building upon this initially promising evidence, our objective is to rigorously examine the effectiveness of FIRST in real-world clinical practice settings. Specifically, an RCET is being conducted to test FIRST relative to UC among ethnically and socioeconomically diverse youths (ages 7–15) who are referred through typical community channels, with treatment delivered by community clinicians (not research employees) in service clinics. These data are needed to determine whether FIRST can enhance youth outcomes in everyday practice when compared to usual care. In addition, the study will examine the role of a promising candidate mediator of change—the regulation of negative emotions—as an initial step in the search for a mechanism of change; the study will also explore implementation variables that are theorized to influence the adoption and effective use of ESTs in community settings.

## Methods

### Aims

The aims and specific research questions of the current trial are as follows:

1. To evaluate the effectiveness of FIRST compared to UC in community mental health clinics.
  - Does FIRST produce faster improvements (i.e., steeper slopes), compared to UC, on measures of internalizing, externalizing, total, and functional top problems during treatment and longer-term through 18 months?
  - Does FIRST produce greater reductions, compared to UC, in total number of mental health diagnoses from pre- to post-treatment?
2. To test a transdiagnostic candidate mechanism of change: regulation of negative emotions.
  a Does FIRST lead to faster improvements in regulation of negative emotions relative to UC?
  b Does improved regulation of negative emotions predict faster improvements in youth clinical outcomes?
  c Does improved regulation of negative emotions mediate the effects of condition (FIRST vs. UC) on youth clinical outcomes?
3. To explore implementation variables specific to clinicians.
  - Do clinician characteristics (e.g., EST knowledge, attitudes, and intentions; perceived organizational climate) predict implementation fidelity, as assessed through observational coding of therapy sessions?
  - Do clinician characteristics (e.g., EST knowledge, attitudes, and intentions; perceived organizational climate) moderate differences in implementation of EST practices between FIRST and UC, including adherence and competence in the delivery of ESTs?

### Design

This study is a multi-site RCET evaluating the superiority of FIRST compared to UC in community outpatient clinics. The design employs single-masked evaluation on all measures (i.e., assessors are naïve to youths’ treatment condition). Allocation to treatment conditions entails masked, sequential, blocked double-randomization (i.e., of both clinicians and youths) within clinic using a computerized, sequential-block approach custom-built for the study with a 1:1 ratio (more details below). The concealment of allocation (until the time of condition assignment and beyond) is ensured primarily by restricting access to the randomization information via password-protected files and secure servers/logins (no one other than the project directors have access to randomization information). The random allocation programs were designed so that it is impossible to know the condition to which future participants will be assigned, even for the person performing the randomization. This concealment is further supported through masking of research staff and data analysts through all phases of the study and by practices meant to eliminate risk for disclosure of allocation (e.g., study protocols are paper-free, so as not to conceal allocation using methods like envelopes, which could be held up to the light or opened and resealed).

All clinicians working at participating clinics (and new clinicians starting at the clinics during the study) are invited to participate and provide written informed consent. Upon enrolling, clinicians are randomly allocated to 1 of 2 conditions: the FIRST condition (i.e., to receive training and other implementation supports in FIRST; see “Intervention Condition: FIRST”) or the UC condition (i.e., to continue providing the treatment they ordinarily provide and believe to be effective; see “Control Condition: Usual Care”). Youth participants who seek treatment via typical referral processes at partner clinics are invited to participate in the study and screened for eligibility by study staff prior to obtaining informed consent from caregivers and assent from participating youths. Youths are then randomly allocated to condition (FIRST vs. UC). To avoid obstructing youths’ access to appropriate care, and to guard against the possibility of important condition differences emerging by chance, a few stratifications and constraints are employed. Random allocation is programed to achieve, approximately, (a) a balance of clinicians’ language fluency (English-only vs. Spanish-bilingual) and education level (doctoral vs. non-doctoral) across conditions; (b) a balance of youth age (younger [7–11] vs. older [12–15]) across conditions; and (c) a ~ 1:1 allocation ratio within each clinical site, for both clinicians and youths.

### Study setting

This study is being conducted in outpatient mental health clinics serving youths in New England and Texas. These clinics have partnered with the research study team and are deemed “clinic partners.” Study procedures were developed in conjunction with clinic partners to ensure that the study did not disrupt normal clinical operations and did not adversely affect families’ access to treatment.

### Participants

#### Youths and caregivers

Youth participants include youths (ages 7–15 at the time of caregiver consent) and their caregivers (used to refer to parents or legal guardians who may or may not be parents) who are seeking mental health treatment at a clinic partner site.

#### Inclusion criteria

Youths are eligible to participate if:

- They are newly referred for treatment at a partner clinic;
- They are between 7.0 and 15.9 years of age on the date of caregiver consent;
- They have at least one scale on the Child Behavior Checklist (CBCL) [30] that is clinically elevated (borderline or clinical range) for symptoms of anxiety, depression, conduct problems, or post-traumatic stress per caregiver report; and,
- They are fluent in English and their caregiver is fluent in English or Spanish.

#### Exclusion criteria

Youths are ineligible to participate if:

- They have current suicide risk (i.e., active suicidal ideation with a plan, or a history of suicide attempt or hospitalization for suicide risk within the last 3 months); or,
- They have a diagnosis of an eating disorder, schizophrenia spectrum disorder, Autism Spectrum Disorder or intellectual disability requiring special class placement in school; or,
- They are referred for the treatment of ADHD specifically and exclusively to address inattentiveness and/or hyperactivity-impulsivity (youths with ADHD symptoms that have elevations in the areas of anxiety, depression, conduct problems, or stress problems on the CBCL are eligible).

Partner clinics identify potential participants during their standard initial intake assessment based on age (7-15) and disqualifying criteria (e.g., suicidality; defined above). Families considered potentially eligible are asked, following a scripted invitation, if the research team may contact the family with study information. If the caregiver gives permission, the research team contacts the family via phone to assess eligibility and provide information about the study. For families who agree to participate, caregivers provide written informed consent and youths provide written assent digitally. To provide fair compensation to families for their time and to incentivize participation and retention, youth and caregivers are compensated for their time completing study measures.

Participants can request to end participation in the study at any time. If participants experience increased risk of suicidal or homicidal behaviors during their participation, and partner clinics and clinicians determine a higher level of care is needed, research participation may be ended to ensure access to an appropriate level of care. Adverse events that are caused by the study are reported as “New Reportable Information” to the IRB. Following IRB review, the study team devises a plan to prevent future adverse events of the same nature. Data monitoring and safety is managed by the project management team. The project management team conducts weekly audits to ensure data integrity (e.g., tracking missing data), and bi-weekly to discuss processes related to enrollment, consent, eligibility, and any reports of potential harms to be reported to the IRB. Additional oversight is conducted annually and as needed by the Data Safety and Monitoring Board (see, Additional File, DSMB Charter for FIRST Trial).

#### Clinicians

Participants include clinicians employed by partner clinics who provide outpatient mental health services to children and adolescents. All clinicians who typically provide care in these partner clinics are eligible to participate regardless of their discipline (e.g., psychology, social work, counseling) or professional level (e.g., licensed practitioners, trainees). Partner clinic administrators facilitate communication between study staff and their clinical staff who are eligible to participate. Study staff explain that participating clinicians will be randomized to either the FIRST condition (and thus receive training immediately) or to the UC condition (where they have an opportunity to receive FIRST training at the end of the study). Interested clinicians are sent an electronic consent form.

#### Intervention condition: FIRST

The FIRST treatment condition includes: 1) use of the FIRST treatment manual, 2) an 18-h training on the content of FIRST and its use with youths 3) weekly small group consultation with FIRST experts to support ongoing fidelity to the treatment, 4) an online learning platform with FIRST resources and 5) an online measurement feedback system that gives clinicians near-real-time data on youth treatment response. The FIRST manual includes skill units (brief one to three page outlines) in the five principles of change (i.e., Feeling Calm, Increasing Motivation, Repairing Thoughts, Solving Problems, and Trying the Opposite) accompanied by worksheets and handouts for youths and their caregivers, as well as skill units specific to Beginning Treatment (e.g., goal setting, engagement, psychoeducation), Ending Treatment (e.g., relapse prevention, termination), and Continuing Treatment/Boosting Engagement (e.g., motivational interviewing strategies to increase engagement in treatment). The manual also includes session content recommendations, in-session activities, and out-of-session assignments related to each principle. The FIRST protocol is designed to be administered to youths, caregivers, and youth-caregiver combinations. FIRST also includes four flowcharts (based on the primary problem) to guide clinical decision-making. The training is a presentation of the manual content that uses experiential activities (video demonstrations, live modeling, and role play) to support learning, and includes time for questions, answers, and discussion. Weekly small group consultation is provided virtually and in-person. Group consultation involves review of performance feedback data for each youth, discussion of the prior session, and planning and rehearsing for the next steps of treatment. Clinicians also have access to an online learning platform with FIRST resources, including the 18-h initial training, additional video demonstrations of how to use FIRST principles with youths and caregivers, worksheets and handouts, and a discussion board monitored by the study team. Finally, the online measurement feedback system provides clinicians with information regarding clients’ symptoms and functional problems on a weekly basis (assessed weekly via the Idiographic Functional Top Problems Assessment and the Behavior and Feelings Survey; see “Primary Clinical Outcomes”). These data are used to monitor response to treatment and guide treatment decisions.

#### Intervention fidelity

FIRST clinicians have weekly consultation where treatment sessions are discussed. Clinicians follow the FIRST manual with the support of a FIRST expert. All sessions (FIRST and UC) are recorded, and a subset will be coded for fidelity to the FIRST protocol to determine the extent to which EST content was delivered in each condition. A portion of sessions are coded in near-real-time (within a week) with fidelity results shared with consultants.

#### Control condition: usual care

UC is the treatment that is usually provided to youths at the partner clinic. This involves the standard training or on-boarding procedures provided to all partner clinic clinicians, and the routine supervision provided. Information on UC is collected via clinician surveys and via coding of a subset of session recordings. Clinicians in the UC condition are not provided access to the FIRST treatment manual, the FIRST training, consultation, online support, or the online performance feedback system.

## Measures

The following measures are collected during the trial (see Tables 1 and 2). Primary and secondary clinical outcome measures are administered to youth and caregivers weekly and/or at 0, 3, 6, 9, 12, and 18 months, with the exception of diagnostic measures which are only administered at pre- and post-treatment (see Table 1). Clinical measures from youth and caregivers are collected via online surveys or telephone calls in which research team members enter data into Qualtrics. Measures from clinicians are collected online using Qualtrics. All data is stored on a HIPAA-compliant, encrypted shared drive to ensure confidentiality. This shared drive is only accessible to research staff using confidential usernames and passwords. Caregiver measures are available in English and Spanish. When measures did not have previously validated Spanish translations, we followed the gold-standard procedures for translation for cross-cultural research [31]. Procedures involved (1) one bilingual team member translating the measure from English to Spanish; (2) a second bilingual team member who had not seen the original English measure back-translating from Spanish back to English; and (3) a third bilingual team member highlighting discrepancies between the original English and back-translated English versions, the three team members meeting to discuss discrepancies and reach consensus on the Spanish version, with the third team member serving as a tie-breaker as needed. All team members involved with translation were fluent in English and Spanish, and translation procedures were led by a team member with prior experience with these procedures.

**Table 1:** Summary of assessment schedule for youth and caregiver data

Measure	Baseline	During therapy	Quarterly^a^	Post-treatment^b^	Informant
Achenbach Child Behavior Checklist/Youth Self Report	X		X		Caregiver and youth
Behavior and Feelings Survey	X	Weekly	X		Caregiver and youth
Functional Top Problems Assessment	X	Weekly	X		Caregiver and youth
Positive and Negative Affect Schedule	X	Weekly	X		Caregiver and youth
Coping Questionnaire	X	Weekly	X		Caregiver and youth
MINI-KID-P	X			X	Caregiver
UCLA PTSD-RI-5	X		X		Caregiver and youth
Youth Participant Background Form	X				Caregiver
Additional Services	X		X		Caregiver
Therapeutic Alliance Scale for Children/Parents		Monthly		X	Caregiver and youth
Parent and Child Satisfaction Scale				X	Caregiver and youth

^a^The quarterly schedule includes assessments occurring at approximately 0, 3, 6, 9, 12, and 18 months since baseline. ^b^Given that length of treatment is not fixed, the post-treatment assessment co-occurs with whichever quarterly assessment is scheduled after the date of discharge for each youth

**Table 2:** Summary of assessment schedule for clinician data

Measure	Baseline	Post-Training	Weekly	Case Termination	Informant
Weekly Clinician Report of Sessions			X		Clinician
Therapeutic Alliance Quality Rating			X	X	Clinician
Therapist Satisfaction Inventory				X	Clinician
Perceptions of Supervisory Support Scales				X	Clinician
Clinician Participant Background Form	X				Clinician
Knowledge of Evidence-Based Services	X	X^a^			Clinician
Evidence-Based Practice Attitude Scale	X				Clinician
Evidence-Based Treatment Intentions	X				Clinician
TCU Organizational Readiness for Change	X				Clinician

^a^Clinicians randomly assigned to the FIRST condition complete measures following their completion of the FIRST training

### Primary clinical outcomes

#### Achenbach System of Empirically Based Assessment (ASEBA) [30]

Youth emotional and behavioral functioning is assessed by ratings from caregivers on the Child Behavior Checklist (CBCL) and by self-ratings on the Youth Self-Report (YSR). Internalizing, Externalizing, and Total Problems scales provide the primary mental health outcomes assessment. The CBCL and YSR have ample evidence for validity, reliability, and sensitivity to change.

#### Behavior and Feelings Survey (BFS) [32]

Weekly assessment of internalizing, externalizing, and total problems will be assessed by the BFS youth and caregiver forms. In prior studies, both youth and caregiver forms showed factor validity, internal consistency, test–retest reliability, convergent validity in relation to three well-established symptom measures (including the CBCL and the YSR), and sensitivity to change during therapy.

#### Functional Top Problems Assessment (TPA) [33]

The TPA assesses severity ratings for the top three functional problems independently identified by the youth and caregiver as most important to address in treatment. The idiographic TPA evidences excellent reliability, validity, and sensitivity to change during treatment.

### Secondary Outcomes (Including Candidate Mediators)

#### Positive and Negative Affect Schedule-Child/Parent (PANAS-C/P) [34]

The 10-item PANAS-C/P includes scales assessing positive affect (5 items, e.g., joyful, cheerful, happy) and negative affect (5 items, e.g., mad, sad, scared). Items are parallel. The 10 items used for this study were identified through item response theory and were shown to accurately classify diagnostic groups in a clinical sample. These 10 items demonstrated similar validity and reliability to the 27-item version [35].

#### Coping questionnaire [36]

The Coping Questionnaire (CQ) is a measure of youths’ ability to regulate emotions during personally identified upsetting situations. In prior studies, the measure demonstrated strong internal consistency, convergent and discriminant validity, criterion validity, and sensitivity to change during treatment. Prior work also demonstrates that the CQ mediates reductions in anxiety among youths who received treatment.

#### Modified version of the MINI International Neuropsychiatric Interview for Children and Adolescents (MINI-KID-P*)* [37]

The MINI-KID-P is a standardized diagnostic interview conducted with caregivers to assess the following diagnoses: Major Depressive Disorder, Bipolar I Disorder, Bipolar II Disorder, Other Specified Bipolar, Panic Disorder, Agoraphobia, Separation Anxiety Disorder, Social Phobia, Specific Phobia, ADHD, Conduct Disorder, Oppositional Defiant Disorder, Adjustment Disorder, Obsessive Compulsive Disorder, Generalized Anxiety Disorder, and Persistent Depressive Disorder. We excluded eating, substance-related, and neurodevelopmental modules from administration, with approval from the measure developer. Assessment of these diagnoses were excluded for one or more of the following reasons: 1) included in study exclusion criteria, 2) have a low base rate in school-age populations, or 3) are assessed through other measures. For developmental and practical reasons, we used only the parent-report component (no youth-report) to form these diagnoses, as they were used for aggregate research purposes but not as individual clinical diagnoses. The interview generates reliable and valid psychiatric diagnoses in a brief administration time.

#### UCLA PTSD Reaction Index (PTSD-RI-5) [38]

Posttraumatic stress (PTS) symptoms are assessed using the PTSD-RI-5 administered to the child and caregiver separately. The PTSD-RI-5 is a semi-structured interview that assesses a child’s trauma history and related PTS symptoms. Results are used to ascertain PTSD diagnostic status, inform whether PTS should be a focus of treatment, and monitor PTS clinical outcomes. The PTSD-RI-5 has been widely used to assess PTS symptoms in children and adolescents. Various studies have shown evidence of internal consistency, test–retest reliability, and validity relative to both the degree of trauma exposure and to PTSD diagnoses on standardized interviews [39–41].

### Youth participant characteristics

#### Youth participant background form

Demographic and other background characteristics of youths and caregivers (e.g., race, ethnicity, gender, socioeconomic status) are collected via the caregiver on the youth participant background form developed for this study.

#### Additional services

Additional services will be assessed via a newly developed measure based on existing measures (e.g., Services Assessment for Children and Adolescents [42, 43]). In this measure, caregivers report whether youths received any non-study related therapy services since the last assessment, and if so, who provided the service and the treatment target. Caregivers also report youth psychotropic medication use, including start dates, end dates, and the types of medications.

### Measures of treatment process and fidelity

#### Therapeutic Alliance Scale for Children/Parents (TASC-C/P) [44]

The TASC-C/P assesses the quality of the therapeutic alliance with the clinician, as reported by both youths and caregivers. The 7-item TASC-C (child form) has shown good internal consistency and test–retest reliability in samples of clinic-referred youths. Likewise, the 7-item parent measure, TASC-P, has shown good internal consistency and test–retest reliability in samples of parents of clinic-referred youths.

#### Therapy Process Observational Coding System-Alliance Scale (TPOCS-A) [45]

The TPOCS-A is an observational coding system applied to recorded therapy sessions by trained study staff that assesses youth-clinician and caregiver-clinician alliance. Psychometric analyses based on youth and caregiver sessions in community outpatient clinics showed that both youth and parent forms have good inter-rater reliability (with most intraclass correlation coefficients in the 0.50 s and 0.60 s), internal consistency (Cronbach’s α = 0.95), associations with established youth- and parent-report questionnaire measures of alliance, and predictive validity of treatment outcome.

#### Therapy Integrity in Evidence-Based Interventions (TIEBI) [46]

The TIEBI is an observational coding system applied to recorded therapy sessions that assesses the presence/absence of the EST procedures of FIRST, and the competency with which these procedures are delivered (rated from 0 [not present] to 4 [expert]). Mean inter-coder agreement has been shown to be strong for both clinician adherence to FIRST (ICC [1, 1] = 0.87) and clinician competence in delivering FIRST procedures (ICC [1, 1] = 0.88) [28].

#### Weekly clinician report of sessions

The weekly clinician report of session survey was developed for the current study to collect information about each session. Clinicians in both conditions report the date of the session, the individuals present at the session, broad problems targeted in the session (e.g., anxiety, misbehavior), their overall impression of the session, and their perceived alliance with the individuals present in the session (e.g., youth, caregiver). Clinicians in the FIRST condition additionally report the FIRST principles used during the session.

#### Therapeutic Alliance Quality Rating (TAQR) [47]

The TAQR assesses the clinician’s report of therapeutic alliance with the caregiver and youth. The TAQR uses a single item to assess each relationship. The single item TAQR is strongly correlated with a 52-item clinician scale used to rate youth and caregiver alliance [48].

#### Engagement of families in treatment

Clinic records will provide detailed data on aspects of the treatment process related to engagement. These will include percent of scheduled sessions attended, attended on time, cancelled, and missed due to no-show; and, whether or not treatment was terminated as planned with clinician agreement.

### Post-treatment measures

#### Therapist Satisfaction Inventory (TSI) [49]

The TSI is a 16-item clinician-report measure that assesses clinician satisfaction regarding their treatment experience with each youth. Psychometric analyses with 145 youths treated in community clinics by 77 clinicians revealed two psychometrically sound subscales indexing clinicians’ perceptions of treatment effectiveness (α = 0.82) and responsiveness (α = 0.81), with clinicians indicating that an EST was perceived as more effective and responsive than UC.

#### Parent and Child Satisfaction Scales (PCSS) [50]

The PCSS is a parallel caregiver- and youth-report measure of treatment satisfaction. Both versions of this measure have previously shown good to excellent internal consistency and test–retest reliability.

#### Perceptions of Supervisory Support Scale (PSSS) [51]

The PSSS is used to assess clinicians’ perceived support in consultation/supervision over the course of treatment with each participant. It is comprised of three subscales including emotional support, support for client goal achievement, and professional development support. It has shown good content validity and reliability.

### Candidate predictors of implementation

#### Clinician participant background form

Background characteristics of the clinicians (e.g., professional discipline, licensure status, therapeutic orientation) are collected via the clinician background form developed for this study.

#### Knowledge of Evidence Based Services Questionnaire-Short Form (KEBSQ-SF) [52]

The KEBSQ-SF is a 17-item self-report measure collected from clinicians to assess their knowledge of practice elements in empirically supported treatments. The measure generates two separate scores: correct endorsements of elements of ESTs and correct rejections of elements without empirical support. The short form has been found to correlate highly with and perform similarly to the well-validated full form of 40 items [53] and demonstrated adequate internal consistency [52].

#### Evidence-Based Practice Attitudes Scale (EBPAS-15) [54]

Clinicians’ attitudes towards ESTs are assessed using the EBPAS, which yields four subscales: appeal (ESTs are intuitively appealing), requirements (would use EST if required), openness (general openness to innovation), and divergence (perceived divergence between EST and current practices). In a large national sample of clinicians, internal consistency was good for the full measure and for the four subscales [53]. Higher scores on the EBPAS openness subscale show convergent validity with clinician-reported cognitive-behavioral therapy use, whereas higher scores on the EBPAS divergence subscale correlate with clinician-reported use of treatment strategies lacking empirical support [55].

#### Evidence-Based Treatment Intentions (EBTI) [56]

The EBTI measures clinicians’ intentions to adopt ESTs clinically. Internal consistency for the scale was good in two studies with community clinicians [57], and the EBTI has demonstrated convergent validity with a measure assessing EST adoption [56].

#### Texas Christian University Organizational Readiness for Change Scale (TCU-ORC) [58]

The TCU-ORC assesses clinicians’ perceptions of the organizational climate of their clinic. Four scale scores will be derived from clinician ratings on this measure: stress (e.g., perceived strain, stress, and role overload); autonomy (e.g., clinician latitude in working with clients); cohesion (e.g., workplace trust and cooperation); and communication (e.g., receptivity to suggestions from staff and utility of information networks). Sound psychometrics for the measure and for these four subscale scores have been reported.

### Sample size and power

The target sample size is *N* = 212 youths. We used Optimal Design [59] to determine power for the main treatment effects, specifically comparing the rate of change for FIRST vs. UC. Assuming at least 24 providers, an ICC of 0.02 (based on prior RCETs), 5 of 6 repeated measures (quarterly schedule, anticipating some missing data), and standard thresholds for significance (α = 0.05), we determined that a minimum sample of *N* = 180 youth participants was required to achieve adequate power (1-β = 0.80) to detect an effect size (ES) of 0.50 This ES of 0.50 or higher is anticipated for two specific primary outcomes measures—the CBCL [30] collected quarterly and the parent-rated TPA [33] collected weekly. As a transdiagnostic intervention trial covering multiple problem areas and a wide developmental range, broad composite measures are most appropriate as the basis for power estimation. Further, caregivers are considered the primary informants due to higher reliability and validity of caregiver report across all youth ages [30, 33, 60]. The selection of the ES magnitude (e.g., 0.50) was based on synthesizing evidence from three previous open trials of FIRST [8] which ranged from 0.45 to 0.98 and randomized trial research with MATCH [15, 16, 61] which ranged from 0.29 to 0.72. The data for MATCH showed an average ES reduction of 43%. When applied to the FIRST findings, the results predicted an ES of 0.51 for FIRST compared to usual care, thus our ES = 0.50 hypothesis. The estimation of an ES of 0.50 strikes an appropriate balance between Type I and Type II errors, and between cost-effectiveness and scientific assuredness. Further, we anticipate a conservative attrition/data loss rate of 15% will occur between allocation and analyses. Thus, a target sample size of *N* = 212 youths will ensure adequate power for the primary analyses (Aim 1).

Monte Carlo simulations were conducted in R using the simsem package [62] to estimate power for the Aim 2 mediation analyses. These simulations revealed that 180 participants will provide sufficient power to detect whether FIRST can produce a 0.36-*SD* change in the slope of the CBCL/YSR symptom improvement through the indirect effect of change in negative affect regulation.

Analyses of variables that may be associated with EST implementation (Aim 3) are considered exploratory and therefore were not designed to be adequately powered. Further, available power will vary across models. Nonetheless, power for these models was simulated using the simr package in R [63] which found a sample size of 180 to be adequate to detect a standardized regression coefficient as low as 0.25 for clinician-level predictors/moderators of outcomes within the FIRST condition (anticipating ~ 90 youths across 12–20 FIRST clinicians).

### Planned analyses

Prior to analysis, univariate, bivariate, distributional, and missing data characteristics of the data will be examined, and subsequent models will be adjusted (e.g., robust estimators, generalized models, non-parametric approaches) to ensure data-analytic assumptions are met. Baseline data from youths, caregivers, and clinicians will be compared across conditions, and any significant (*p* < 0.05) differences that emerge will be considered as covariates or moderators in subsequent analyses. Medication use, gender, and race/ethnicity may be examined as covariates and moderators in all outcome models to assess the robustness of effects. Missing data will be accommodated using full information maximum likelihood estimation or multiple imputation. Due to the low risk nature of this intervention, there will be no interim analyses; all data analyses will be conducted when data collection is complete.

#### Aim 1

The effectiveness of FIRST compared to UC will be assessed using longitudinal multilevel models estimated with repeated measures at level 1 (5 + observations; random effects) nested within participants at level 2 (*N* = 180, random effects) nested within clinicians at level 3 (*N* = 24; random effects). Clinics will be modeled via dummy-coded fixed effects at level 3 and time will be modeled as linear or log-linear days since baseline. The effectiveness of FIRST vs. UC will be tested via the contrast term for the slopes of change in each condition. A significant (*p* < 0.05) contrast indicates that one group improved more rapidly than the other. The magnitude of this effect will be examined through the standardized mean difference effect size of the slope contrast (i.e., difference between the two conditions’ slopes divided by the square root of the overall slope variance), with clinical significance examined in terms of model-implied outcomes at 6, 12, and 18 months within each group. To complement these primary symptom trajectory outcomes, we will also examine change in total number of diagnoses on the modified MINI-KID-P at post-treatment using generalized linear models, controlling for baseline diagnoses.

#### Aim 2

Latent growth curve (LGC) mediation models will be estimated to test measures of regulation of negative emotions as a candidate mechanism of change. Mediation will be specified using the randomly assigned intervention condition (FIRST vs. UC) as an observed variable, and latent intercepts (baseline levels) and slopes (linear change) in primary and secondary outcomes based on multiple occasions of measurement (e.g., weekly, 0–3-6–9-12–18 months). The LGC models will allow for assessment of whether changes in negative affect (e.g., reduction in negative emotions, increases in coping) that occur early on (e.g., weekly during treatment, or 0–3-6 months) account for symptom change that occur in the long-term (e.g., CBCL/YSR outcome trajectories through 18 months). In this way, these models will achieve the requisite temporal sequence to establish that a variable actually functions as a mechanism of change for mediation.

#### Aim 3

Aim 3 analyses will be conducted in an exploratory manner. Data collected from clinicians at baseline in both conditions will be examined in multilevel regression models as level-2 (clinician-level) predictors of level-1 (youth-level) implementation outcomes (e.g., TIEBI-coded adherence and competence). For these models, we will use case-level mean TIEBI scores (i.e., averaging across beginning, middle, and end of treatment scores for each youth) as the outcome variables. As the predictors, we will use scores for measures of clinicians’ baseline knowledge of (KEBSQ), attitudes toward ESTs (EBPAS), motivation to use ESTs (EBTI), and perceptions of organizational climate. Models will be estimated in a hierarchical two-step sequence. In step 1, we will examine these four variables as predictors of implementation outcomes. In step 2, we will test whether these four variables interact with treatment condition; this may clarify whether these evidence-based practice psychological variables predict implementation outcomes differently for those in the FIRST condition as compared to UC.

We used the SPIRIT checklist when writing our report [64].

## Discussion

This study is the first RCET of FIRST—a principle-based transdiagnostic treatment built on five ESPCs. FIRST has shown promising effects across three open benchmarking trials [8, 29]. Study results will indicate the effectiveness of FIRST, compared to usual outpatient care, in reducing internalizing, externalizing, total problems, and idiographic functional top problems among youths in community mental health settings. Additionally, this study will test the regulation of negative emotions as a candidate mediator of change and explore factors that may affect implementation. A particular strength of this multi-site RCET is the partnership with publicly funded clinics and inclusion of bilingual clinicians, which is likely to result in a racially, ethnically, linguistically, and socioeconomically diverse sample, enhancing the generalizability of its findings.

One potential challenge includes enrolling the required youth sample size. To address this challenge, we will actively recruit additional clinic partners from which we can recruit more clinician and youth participants if needed. Another potential challenge is clinician turnover, which is anticipated as participating clinicians leave partner clinics or shift to administrative positions. Thus, we plan to recruit clinicians from partner clinics on a rolling basis, randomizing them to condition and providing FIRST training to clinicians allocated to FIRST throughout the active enrollment phase of the trial.

Results may shed light on a next-generation treatment approach to personalizing youth mental health care. Positive results may help increase support for widespread implementation of transdiagnostic, principle-based treatments like FIRST, which seek to address many of the obstacles to youth EST delivery in real-world practice contexts (e.g., narrow problem coverage, youth comorbidity, limited flexibility). Findings from this study will also indicate this treatment’s ability to provide symptom improvement across a range of comorbid internalizing and externalizing problems via a promising transdiagnostic mechanism of change: the regulation of negative emotions. This study will inform whether FIRST, which combines five ESPCs, could enhance youths’ capacity to regulate negative emotions, and whether symptom reduction might be mediated by improved regulation. Additionally, findings may elucidate clinician factors (e.g., EST knowledge, attitudes, and intentions; organizational climate) that may be associated with FIRST implementation and outcomes, and therefore may reveal the potential for more efficient, principle-guided interventions to address the implementation cliff.

### Trial status

The Harvard University Institutional Review Board has approved the study procedures. The protocol has been revised 17 times and was reviewed in July 2023. Recruitment of clinicians began in July 2021 and recruitment of youths began in September 2021. Recruitment will end in 2025.

### Supplementary Information

**Additional file 1.** Caregiver consent  for child to take part in a human research study.

**Additional file 2.** Consentimiento para tomar parte en un estudio de investigación de los humanos.

**Additional file 3.** Assent to take part in a human research study.

**Additional file 4.** Clinician consent to take part in a human research study.

**Additional file 5. **SPIRIT guidelines checklist.

## Data Availability

The final trial data set will be maintained by the principal investigators. Following acceptance for publication of the primary analyses, the dataset will be available via the NIMH Data Archive (NDA).

## Abbreviations

BFS: Behavior and Feelings Survey
CBCL: Child Behavior Checklist
CQ: Coping Questionnaire
EBPAS: Evidence-Based Practice Attitudes Scale
EBTI: Evidence-Based Treatment Intentions
ESPC: Empirically supported principles of change
EST: Empirically Supported Treatment
FIRST: Principle-Guided Psychotherapy for Children and Adolescents: The FIRST Program
KEBSQ: Knowledge of Evidence Based Services Questionnaire
LGC: Latent growth curve
MATCH: Modular Approach to Therapy for Children with Anxiety, Depression, Trauma, and Conduct
MINI-KID-P: Modified version of the MINI International Neuropsychiatric Interview for Children and Adolescents
PANAS-C/P: Positive and Negative Affect Schedule-Child/Parent
PCSS: Parent and Child Satisfaction Scales
PSSS: Perceptions of Supervisory Support Scale
PTSD-RI-5: UCLA PTSD Reaction Index
RCET: Randomized controlled effectiveness trial
TASC-C/P: Therapeutic Alliance Scale for Children/Parents
TCU-ORC: Texas Christian University Organizational Readiness for Change Scale
TIEBI: Therapist Integrity in Evidence-Based Interventions
TPA: Functional Top Problems Assessment
TPOCS-A: Therapy Process Observational Coding System-Alliance Scale
TSI: Therapist Satisfaction Inventory
UC: Usual care
YSR: Youth Self-Report
