# Supplementary material for: Principle-Guided Psychotherapy for Children and Adolescents (FIRST): study protocol for a randomized controlled effectiveness trial in outpatient clinics
Source: Trials. 2023 Oct 21;24:682. doi: 10.1186/s13063-023-07717-y (PMC10589969; doi:10.1186/s13063-023-07717-y)
Supplement: Supplementary file 1 — Additional file 1. Caregiver consent for child to take part in a human research study. [file 13063_2023_7717_MOESM1_ESM.pdf]

Caregiver Permission for Child to Take Part in a Human Research Study

**Protocol Title:** Testing FIRST in Youth Outpatient Psychotherapy
**Principal Investigator:** John R. Weisz, Ph.D.
**Description of Study Population:** Families (youths ages 7-15 and their caregivers) seeking community-based mental health services for anxiety, depression, trauma, or misconduct.
**Version Date:** 9.24.21

Key Information

Your child is being invited to participate in a research study directed by Dr. John Weisz, a professor and researcher at Harvard. More detailed information is provided later in this form.

Why is my child being invited to take part in a research study?

We have invited your child to take part in this research study because your family is seeking mental health services for your child at a local community mental health clinic. We are seeking your permission, as a caregiver (i.e., parent or legal guardian), for your child to participate in this research study.

What should I know about a research study?

- A member of our research team will explain this research study to you/your child.
- Whether or not you/your child take part is voluntary. It is your choice whether to participate and whether to permit your child to participate.
- You/your child can choose not to take part in the study. If you/your child disagree on whether to take part, you can discuss this before deciding. If you still do not agree, your family will not be enrolled in the research study.
- You/your child can agree to take part and later change your mind.
- If you/your child choose not to participate or later decide to withdraw from the study, this decision will not be held against you/your child. There will be no penalty or loss of benefits to which you/your child are otherwise entitled. If your child indicates a wish to withdraw from the study, you can discuss this decision with your child. If you do not agree, your family's participation in the research study will be discontinued.
- You/your child can ask all the questions you want before making a decision.

Why is this research being done?

The purpose of the study is to test the effectiveness of two different mental health treatment approaches for problems related to anxiety, depression, trauma, or misbehavior in children and adolescents. In **Treatment #1**, therapists at your local community mental health clinic are trained and receive consulting in the use of a new mental health treatment approach shown to be effective elsewhere and now being used in your local clinic. In **Treatment #2**, therapists at your local community mental health clinic rely on their previous training and experience. Some families in the research study will receive Treatment #1, and some families will receive Treatment #2. Which treatment your family receives is chosen randomly (like the flip of a coin). Therapists using both treatment approaches will be doing their very best to help their clients. The study will help us to understand how helpful the new mental health treatment approach is. To help us answer this question, we are also inviting your child to participate in several assessments. We will pay your child for completing these assessments. Your child could earn up to $175.

How long will my child take part in this research?

The duration of the mental health treatment will be decided by your child's therapist. The assessments will take place for a total of 18 months—some on a weekly basis and others occurring roughly every 3 months (i.e., at 0, 3, 6, 9, 12, and 18 months)—beginning when your child starts therapy.

What will my child's participation in the study involve?

You will complete an initial phone screen to determine if your child is a good fit for the study. If your child is eligible for the study, the next phase of the study involves therapy sessions at your local mental health clinic. Your child will also be asked to participate in regular assessments: one before your child's first therapy session. After this, some shorter assessments will occur weekly while your child is in therapy, and other longer assessments will take place roughly every three months (i.e., at 3, 6, 9, 12, and 18 months) after your child's starts the study. Assessments will be conducted online or over the phone with a member of the research team at Harvard—at a time that is convenient for your family. Your child will be paid for completing the assessments. Additionally, we will ask for your consent to allow us to access your child's records from your local community mental health clinic. Therapy will be provided by therapists from your local community mental health clinic who have experience working with children, adolescents, caregivers, and families. We will ask for your and your child's permission to audio-record or video-record these therapy sessions, which is required for participation in the study. Assessments and audio- or video-recordings will be used by the research team and will not be part of your child's mental health records. More detailed information about the study procedures can be found under the *"What can I expect if I take part in this research*?" section.

Is there any way being in this study could be bad for my child?

Your child may grow tired from the assessment questions, and some of the questions may ask about topics that your child would rather not discuss. Your child may choose not to answer any question and may stop any assessment at any time. More detailed information about the risks of this study can be found under the *"What are the risks and possible discomforts?"* section.

Will being in this study help my child in any way?

We cannot promise any benefits to your child from taking part in this research. However, therapy is designed to be helpful to children and adolescents with problems related to anxiety, depression, trauma, or misbehavior. It is also possible that your child may find it helpful to talk with members of the research team during assessments. Information from this study may also be helpful to families in the future by contributing to improved youth mental health services.

What happens if my child does not want to be in this research?

Participation in research is completely voluntary. You and your child can decide to participate or not to participate. This decision will not impact the services your child is otherwise entitled to receive. If you and your child disagree on whether to take part, you can discuss this before deciding. If you still do not agree, your family will not be enrolled in the research study.

Detailed Information

To follow, please find more detailed information about this study than already provided above.

About this permission form:

Please read this form carefully. It provides important information about participating in research. You have the right to take your time in making decisions about your child's participation in this research. If you have any questions about the research or any portion of this form, you can ask us at any time. If you decide to permit your child to participate in this research study, you will be asked to sign this form. A copy of the signed form will be provided to you for your record.

Who can I talk to?

If you have questions, concerns, or complaints—or think the research has hurt your child—Dr. John Weisz, the principal investigator of this study, can be reached at Harvard University, 1030 William James Hall, 33 Kirkland Street, Cambridge, MA 02138 or by email at john_weisz@harvard.edu. This research study has been reviewed by the Committee on the Use of Human Subjects (CUHS) at Harvard University. If you wish to speak to a representative from the IRB, you may contact (617)-496-2847 or cuhs@harvard.edu for any of the following:

- If your/your child's questions, concerns, or complaints are not being answered by the research team.
- If you/your child cannot reach the research team.
- If you/your child want to talk to someone besides the research team.
- If you/your child have questions about rights as research participants.
- If you/your child want to get information or provide input about this research.

Participation is voluntary.

Your child is being invited to take part in this research study because your family is seeking mental health services for your child at a local community mental health clinic. It is your choice whether to permit your child to participate. If you and your child disagree on whether to take part, you can discuss this before deciding. If you still do not agree, your family will not be enrolled in the research study. If you choose to allow your child to participate, you or your child may change your mind and leave the study at any time. If your child indicates a wish to withdraw from the study, you can discuss this decision with your child. If you do not agree, your family's participation in the research study will be discontinued. Refusal to participate or stopping participation will involve no penalty or loss of benefits to which your child is otherwise entitled. This decision will not impact the mental health services your child receives.

How many people will take part in this research?

Approximately 210 families and 40 therapists are expected to take part in this research study.

What can I expect if my child takes part in this research?

As a participant, your child will be expected to complete the following. If your family is eligible for the study (based on an initial phone screen with you), your child will be asked to complete an initial assessment before their first therapy session. This assessment will last 60 minutes, and it will be conducted over the phone with a study staff member. The next phase of the study involves therapy sessions at your local community mental health clinic. During this phase of the study, your child will be asked to participate in regular assessments. Some of these assessments will occur weekly, and others will take place about every three months. These quarterly assessments will continue for a total of 18 months after your child begins the study (i.e., at about 3, 6, 9, 12 and 18 months after your child starts the study).

All assessments will be conducted online or over the phone with a member of the research team at Harvard—at a time that is convenient for your family. Weekly assessments with your child will take no more than ten minutes, and quarterly assessments will last 30-60 minutes. Your child will be paid for completing the assessments. Additionally, we will ask for your consent to allow us to access your child's records from your local community mental health clinic. Therapy will be provided by therapists from your local community mental health clinic who have experience working with children, adolescents, caregivers, and families. We will ask for your consent and your child's permission to audio-record or video-record these therapy sessions, which is required for participation in the study. Assessments and recordings will be used by the research team and will not be part of your child's mental health records.

What are the risks and possible discomforts to my child?
Your child may grow tired from the assessment questions, and some of the questions may ask about topics that your child would rather not discuss. Your child may choose not to answer any question and may stop any assessment at any time. There is also the possibility that your child's confidentiality may be breached; however, we will take great efforts to protect the privacy of your child and thus to minimize this risk.

Are there any benefits to my child from being in this research study?
We cannot promise any benefits to your child from taking part in this research. However, the therapy approaches used in this study are designed to be helpful to children and adolescents with problems related to anxiety, depression trauma, or misbehavior. It is also possible that your child may find it helpful to talk with members of the research team during assessments. Information from this study may also be helpful to families in the future by contributing to improved youth mental health services.

What happens if I give my permission, but I change my mind later?
Your child can leave the research study at any time. This decision will not impact the services your child receives. If your child indicates a wish to withdraw from the study, you can discuss this decision with your child. If you and your child do not agree, your family's participation in the research study will be discontinued. If your child decides to discontinue participation (or you withdraw your permission), we may ask for your permission to include data previously collected from your child in our analysis.

Can my child still receive mental health services at our local community mental health clinic if I do not allow my child to participate in this research?
Yes, your child can continue to receive mental health services at your local community mental health clinic if you choose not to give permission. Your decision will not change the care your child receives now or in the future. If you give permission for your child to take part in this study, your child may leave or stop the study at any time. There will be no penalty to your child, and your child's mental health care will not be affected. If your child would like to stop participating in this study (or you would like to withdraw your permission), please let a member of the research team know immediately.

Will my child be compensated for participating in this research?
Your child will be compensated for completing weekly and quarterly (i.e., roughly every 3 months) research assessments. The table below summarizes compensation for each assessment.

Type of Research Assessment	Caregiver Est. Time	Caregiver Payment	Child Est. Time	Child Payment
Initial assessment	90 minutes	$30	60 minutes	$10
Weekly assessments	10 minutes	$5	10 minutes	$2.50

3-month assessment	60 minutes	$30	30-60 minutes	$10
6-, 9-month assessments	60 minutes	$40	30-60 minutes	$15
12-month assessments	60 minutes	$50	30-60 minutes	$20
18-month assessment	60 minutes	$60	30-60 minutes	$25

What will I have to pay for if I permit my child to participate in this research?
You will pay for therapy sessions at your local mental health clinic under the usual circumstances and costs of families seeking therapy services there (e.g., reimbursed by Medicaid or private insurance). We will compensate clinics for participating in the research study to ensure that they do not lose revenue or staff time (e.g., being trained or participating in consultations) that would undermine their usual services to their communities. If your family is assigned to new treatment approach (Treatment #1), you will incur no additional costs compared to accessing your clinic's usual therapy services.

If my child takes part in this research, how will their privacy be protected? What happens to the information you collect?
Every effort will be made to limit the use and disclosure of your child's personal information, including research study and mental health records, to people who have a need to review this information, including representatives from the IRB. Only researchers will ever have access to your child's information—and only for the purposes to which you and your child agree. We will use coded identification numbers on all electronic files and audio-recordings/video-recordings, and these computerized and digital data will be stored on secure and password-protected computers, platforms, and networks. To permit us to contact you and your child, we will keep your contact information in a password-protected computer file, separate from your child's research data. Any other documents that have potentially identifying data will be stored separately as well. Data from this study will be valuable sources of information about therapy for youth in community-based mental health clinics. Therefore, we will keep study data indefinitely, using and sharing it—in de-identified form only—for research purposes. We will not share any information that could identify your child. The use and sharing of study data will be overseen by a committee whose role is to ensure that the rights of your child as a research participant are protected. Audio-recorded and video-recorded data will be kept until the study is complete and all study information has been fully analyzed. After that, the recordings will be permanently deleted (unless you and your child give us separate/explicit permission for the audio-recordings or video-recordings to be kept for educational/training purposes).

Are the any exceptions to confidentiality?
Confidentiality does not extend to information about possible child abuse/neglect or significant risk of harm to self or others. If our research team is given such information, we are required by law to take necessary actions in order to protect you, your child, or others from harm. This may include reporting these risks to senior members of the research team, appropriate authorities, your child's clinician, or any person who might be in danger.

What else should I know about confidentiality?
A description of this study will be available on http://www.ClinicalTrials.gov, as required by U.S. Law. This website will not include information that can identify your child. At most, the website will include a summary of the results. You can search this website at any time. To help us protect your

child's privacy, this research is covered by a Certificate of Confidentiality from the National Institutes of Health. The researchers can use this Certificate to legally refuse to disclose information that may identify your child in any federal, state, or local civil, criminal, administrative, legislative, or other proceedings (for example, if there is a court subpoena). The Certificate of Confidentiality will not be used to prevent disclosure to state or local authorities of any information receive about possible child abuse or risk for harm to self or others.

What else do I need to know?
This research is being funded by the National Institutes of Health. Drs. Weisz and Bearman, the principal investigators of this research study, are professors at Harvard University and University of Texas at Austin, respectively, which require all faculty to disclose financial information in research study consent forms. Drs. Weisz and Bearman are authors and editors of multiple treatment manuals and books on therapy for children, adolescents, and families, and they are eligible to receive royalties from their publishers. Thus, they can receive some income from publishers if the treatment approaches they have written about are successful and other people use them. Please ask any questions that you or your child might have about this.

Recordings for Educational and Training Purposes
As mentioned previously, your child's therapy sessions will be audio-recorded or video-recorded. These recordings will be used for research purposes only, and your family's identity will be kept confidential. Once the study is complete and all study information has been collected and analyzed, these recordings will be permanently deleted. However, if you provide us with permission to retain the audio-recordings or video-recordings after the study, they will be only used for educational and training purposes. Your family's identity will remain confidential. Your willingness to allow us to keep these recordings will not affect your family' ability to participate in this study nor will it affect the mental health services your child receives. Please indicate your choice below:

☐ **Yes, I willingly give permission** for the research team to retain and use *video-recordings* of my child's therapy sessions for educational or training purposes beyond the completion of this study.

☐ **Yes, I willingly give permission** for the research team to retain and use *audio-recordings* of my child's therapy sessions for educational or training purposes beyond the completion of this study.

☐ **No, I do not give permission** for the research team to retain and use video-recordings or audio-recordings of my child's therapy sessions for educational or training purposes beyond the completion of this study.

Use of Mental Health Clinic Records for Research Purposes
For the purposes of this study, we are requesting access to your child's records from your local community mental health clinic. Access to your child's clinic records **are required** for your child to participate in this study.

☐ **Yes, I willingly give permission** for the research team to use and retain my child's clinic records for research purposes.

☐ **No, I do not give permission** for the research team to use and retain my child's clinic records for research purposes.

Caregiver Permission for Child to Take Part in a Human Research Study

Statement of Permission
I have read the information in this permission form including risks and possible benefits. All my questions about the research have been answered to my satisfaction. I understand that I am free to withdraw my permission at any time without penalty or loss of benefits to which my child is otherwise entitled.

I agree for my child to participate in the study.

Caregiver Permission

Your signature below indicates your permission for your child to take part in this research.

____
Name of child participant

____ __________________________
Signature of first caregiver (parent or guardian) Date

- ❑ Parent
- ❑ Guardian (see note below)

____
Name of first caregiver (parent or guardian)

____ __________________________
Signature of second caregiver (parent or guardian) Date

- ❑ Parent
- ❑ Guardian (see note below)

____
Name of second caregiver (parent or guardian)

If signature of second caregiver (parent or guardian) not obtained, indicate why: (select one)

- ❑ IRB determined that the permission of one caregiver (parent or guardian) is sufficient
- ❑ Second caregiver (parent or guardian) parent is deceased
- ❑ Second caregiver (parent or guardian) is unknown
- ❑ Second caregiver (parent or guardian) is incompetent
- ❑ Second caregiver (parent or guardian) is not reasonably available
- ❑ Only one caregiver (parent or guardian) has legal responsibility for the care and custody of the child

Assent

- ❑ Obtained
- ❑ Not obtained because:
  - ❑ IRB determined that assent of the child was not a requirement
  - ❑ The capability of the child is so limited that the child cannot reasonably be consulted.

____
Signature of person obtaining consent and assent

____ __________________________
Name of person obtaining consent and assent Date

Caregiver Permission for Child to Take Part in a Human Research Study

[*or*, in cases where clinic partners agree that it is not feasible to obtain a digital signature]

By selecting yes below, you are indicating that you understand what you just read, that we answered your questions, and that you agree to permit your child to participate in this research project.

☐ **Yes, I agree to permit my child to particiate in the study.**

☐ **No, I do not agree to permit my child to participate in the study.**
