# Supplementary material for: Principle-Guided Psychotherapy for Children and Adolescents (FIRST): study protocol for a randomized controlled effectiveness trial in outpatient clinics
Source: Trials. 2023 Oct 21;24:682. doi: 10.1186/s13063-023-07717-y (PMC10589969; doi:10.1186/s13063-023-07717-y)
Supplement: Supplementary file 2 — Additional file 2. Consentimiento para tomar parte en un estudio de investigación de los humanos. [file 13063_2023_7717_MOESM2_ESM.pdf]

Consentimiento para Tomar Parte en un Estudio de Investigación de los Humanos

**Título del Protocolo:** Probando FIRST en la Psicoterapia Ambulatoria de Jóvenes
**Investigador Principal:** John R. Weisz, Ph.D.
**Descripción de la Población del Estudio:** Familias (jóvenes de 8-15 años y sus cuidadores/as) buscando servicios comunitarios de salud mental para la ansiedad, la depresión, el trauma o la mala conducta.
**Fecha de la Versión:** 8-27-2021

Información Importante

Usted y su hijo/hija están invitados/as a participar en un estudio de investigación dirigido por Dr. John Weisz, un profesor e investigador de Harvard. Información más detallada sobre el estudio se podrá encontrar en esta forma.

¿Por qué me han invitado a participar en un estudio de investigación?

Nosotros le/la hemos invitado a usted (como cuidador/cuidadora—es decir, como padre o tutor legal) y a su hijo/a a participar en este estudio de investigación porque usted está buscando servicios de salud mental para su hijo/a en la clínica local comunitario de salud mental

¿Qué debería saber sobre un estudio de investigación?

- Un miembro de nuestro equipo de investigación le explicara este estudio en particular a usted y a su hijo/a.
- Es voluntario que usted y su hijo/hija participe. Es su decisión participar y permitir que su hijo/a participe.
- Puede decidir no participar en este estudio. Si usted y su hijo/a no están de acuerdo en la decisión de formar parte en este estudio o no, lo pueden discutir antes de decidir. Si todavía no están de acuerdo, su familia no será inscrito en la investigación.
- Puedes decidir participar en el estudio y luego cambiar de opinión.
- Si decide no participar o decide retirar del estudio más tarde, su decisión no será usado en contra de usted o su hijo/a. No habrá ninguna penalización o perdida de beneficios que por lo demás ya usted o su hijo/a tenga el derecho a acceder. Si su hijo/a desea retirarse del estudio, usted puede discutir esta decisión con su hijo/a. Si no están de acuerdo, la participación de su familia en la investigación será descontinuada.
- Usted y su hijo/a puede hacer todas las preguntas que quiera antes de decidir.

¿Por qué se está haciendo esta investigación?

El propósito de este estudio es determinar que tan efectivo son dos distintos enfoques de tratamiento para la salud mental con relación a problemas con la ansiedad, la depresión, el trauma o la falta de conducta en jóvenes y adolescentes. En el **Tratamiento #1**, terapeutas en su clínica local comunitario de salud mental son entrenados/as y reciben consulta en el uso de un nuevo tipo de enfoque de tratamiento para la salud mental que se ha demostrado a ser efectivo en otras clínicas y ahora se está implementando en su clínica local. En el **Tratamiento #2**, terapeutas en su clínica local comunitario de salud mental solamente dependerán de sus entrenamientos y experiencias anteriores. Algunas familias en la investigación recibirán el Tratamiento #1, y otras familias recibirán el Tratamiento #2. El tratamiento que recibirá su familia será determinado de manera aleatoria (como lanzando una moneda). Terapeutas usando ambos enfoques de tratamiento harán su mejor esfuerzo para ayudar a sus clientes. Esta investigación nos

ayudara a entender que tan efectivo es el nuevo tipo de tratamiento para la salud mental. Para ayudarnos a responder esta pregunta, también le/la estamos invitando a usted y a su hijo/a a participar en varias evaluaciones. Usted y su hijo/a serán recompensados/as por completar estas evaluaciones. La recompensa que recibe puede aumentar a un total de $410 y su hijo/a puede recibir hasta $175.

¿Por cuánto tiempo formaré parte de esta investigación?

La duración del tratamiento de salud mental será determinada por el/la terapeuta de su hijo/a. Las evaluaciones tomarán lugar por un total de 18 meces—algunos semanalmente y otros ocurriendo aproximadamente cada 3 meces—empezando cuando su hijo/a empieza la terapia. Para más información sobre el tiempo exacto de las evaluaciones trimestrales, lee la sección "**¿Hay recompensa por mi participación en este estudio?"**

¿Qué implicará nuestra participación en el estudio?

Se le pedirá que usted complete una evaluación telefónica inicial para determinar si su familia es adecuada para participar en el estudio. Si usted y su hijo/a son elegibles para el estudio, la próxima fase implicará tomar parte en sesiones de terapia en su clínica local de salud mental. También se les pedirán a usted y su hijo/a que participen en evaluaciones regulares. Algunas de estas evaluaciones ocurrirán semanalmente y otras serán aproximadamente cada tres meses (antes de la primera sesión de terapia de su hijo/a y luego de nuevo a los 3, 6, 9, 12 y 18 meces después de la primera sesión de terapia de su hijo/a). Todas evaluaciones tomarán lugar a través del internet o del teléfono con un miembro del equipo de investigación en Harvard—a una hora conveniente para su familia. Usted y su hijo/a serán recompensados por completar las evaluaciones. También, se le pedirá su consentimiento para acceder a los archivos de su hijo/a en su clínica local comunitario de salud mental . La terapia será proporcionada por los/las terapeutas de su clínica local comunitario de salud mental quienes tienen experiencia trabajando con jóvenes, adolescentes, cuidadores y familias. Se le pedirá su consentimiento para grabar en audio o video estas sesiones de terapias, lo cual es requerido para participar en el estudio. Las evaluaciones y grabaciones serán usados por los investigadores y no formarán parte de los archivos de salud mental de su hijo/a. Información mas detallada sobre el procedimiento del estudio se puede encontrar en la sección *"¿Qué puedo esperar si participo en esta investigación?"*

¿Cuáles son las posibles consecuencias negativos de participar en este estudio?

Usted o su hijo/a pueden cansarse de las preguntas de las evaluaciones, y algunas preguntas podrán preguntar sobre temas de las que quizás no querrán hablar. Usted y su hijo/a pueden decidir no responder cualquiera pregunta y pueden parar cualquiera evaluación a cualquier tiempo. Información mas detallada sobre los riesgos de participar en este estudio se pueden encontrar en la sección *"¿Cuáles son los riesgos y posibles incomodidades?"*

¿Cuáles son los posibles beneficios de participar en este estudio?

No se les puede prometer a usted y su hijo/a que habrá beneficios por participar en este estudio. Sin embargo, la terapia es diseñada para ayudar a los jóvenes y adolescentes con problemas relacionados a la ansiedad, la depresión, el trauma o la falta de conducta. También es posible que hablar con los investigadores durante las evaluaciones le ayude a usted o su hijo/a. Además, la información que se obtiene en este estudio puede ayudar a familias en el futuro a través de su contribución al mejoro de servicios de salud mental juveniles.

¿Qué pasa si no quiero participar en este estudio?

Participación en la investigación es completamente voluntario. Usted y su hijo/a pueden decidir participar o no participar. Esta decisión no impactará a los servicios que ya usted o su hijo/a tenga el derecho a recibir. Si usted y su hijo/a no están de acuerdo en la decisión de formar parte en este estudio o no, lo pueden discutir antes de decidir. Si todavía no están de acuerdo, su familia no será inscrito en la investigación.

Información Detallada

Para seguir, abajo encontrará información más detallada sobre el estudio que la proporcionado anteriormente.

Sobre esta forma de consentimiento:

Por favor lea esta forma cuidadosamente. Proporciona información importante sobre la participación en la investigación. Tiene el derecho de tomarse su tiempo en hacer decisiones relacionados con su participación en esta investigación. Si usted tiene cualquiera pregunta sobre la investigación o sobre cualquiera sección de esta forma, nos puede preguntar en cualquier momento. Si decide participar en este estudio de investigación, se le pedirá que firmes esta forma. Una copia de la forma firmada se le dará para sus archivos.

¿Con quién puedo hablar?

Si usted tiene preguntas, preocupaciones o quejas—o cree que la investigación le ha causado daño a usted o su hijo/a—el Dr. John Weisz, el investigador principal de este estudio puede ser contactado en la Universidad Harvard, 1030 William James Hall, 33 Kirkland Street, Cambridge, MA 02138 o a través del correo electrónico: john_weisz@harvard.edu. Este estudio de investigación ha sido revisado por la Comité para el Uso de Sujetos Humanos (CUHS) en la Universidad Harvard. Si quiere hablar con un representante de la Junta de Revisión Institucional (IRB), puede llamar al (617) 496-2847 o cuhs@harvard.edu por cualquiera de las siguientes razones:

- Si sus preguntas, preocupaciones o quejas no han sido abordados por el equipo de investigación.
- Si no ha podido contactar al equipo de investigación.
- Si quiere hablar con alguien que no sea del equipo de investigación.
- Si tiene preguntas sobre sus derechos como participante de la investigación.
- Si quiere obtener información o hacer aportaciones sobre esta investigación.

Su participación es voluntaria.

Usted y su hijo/a han sido invitado a participar en este estudio de investigación porque usted está buscando servicios de salud mental para su hijo/a en la clínica local comunitario de salud mental . Es su decisión si participe y si permite a su hijo/hija a participar. Si decide participar, puede cambiar de opinión y parar el estudio en cualquier momento. Si usted y su hijo/hija no están de acuerdo en su decisión de participar o no, usted tiene tiempo para hablar esta decisión con su hijo/a. Si todavía no están de acuerdo, su familia no será inscrito en la investigación. No habrá ninguna penalización o perdida de beneficios que ya usted o su hijo/a tenga el derecho a acceder si decide no participar o parar el estudio más tarde. Esta decisión tampoco impactará a los servicios de salud mental que recibe su hijo/a. Si su hijo/a desea retirarse del estudio, puede discutir esta decisión con su hijo/a. Si no están de acuerdo, la participación de su familia en la investigación será descontinuada.

Consentimiento para Tomar Parte en un Estudio de Investigación de los Humanos

¿Cuántas personas participarán en este estudio?

Aproximadamente 210 familias y 40 terapeutas son esperados a formar parte de esta investigación.

¿Qué puedo esperar si participo en esta investigación?

Como participante, se le pedirá que hagas lo siguiente. Primero, se le pedirá que complete una evaluación telefónica inicial para determinar si su familia es adecuada para participar en el estudio. Si su familia es elegible y hay un cupo en el estudio, evaluaciones de referencia se harán con usted y su hijo/a individualmente, y se espera que cada uno dure 60-90 minutos. La próxima fase implicará formar parte en sesiones de terapia en su clínica local de salud mental. Durante esta fase del estudio, también se les pedirá que participen en evaluaciones regulares. Algunas de estas evaluaciones ocurrirán semanalmente y otras serán aproximadamente cada tres meses durante un total de 18 meces (a las 3, 6, 9 ,12 y 18 meces). Todas evaluaciones tomarán lugar a través del internet o del teléfono con un miembro del equipo de investigación en Harvard—a una hora conveniente para su familia. Evaluaciones semanales tomarán no más que diez minutos y las mensuales tomarán aproximadamente una hora. Usted y su hijo/a serán recompensados por completar las evaluaciones. Además, se le pedirá su consentimiento para acceder a los archivos de su hijo/a en su clínica local comunitario de salud mental. La terapia será proporcionada por los/las terapeutas de su clínica local comunitario de salud mental quienes tienen experiencia trabajando con jóvenes, adolescentes, cuidadores y familias. Se le pedirá su consentimiento para grabar en audio o video estas sesiones de terapias, lo cual es requerido para participar en el estudio. Las evaluaciones y grabaciones serán usados por los investigadores y no formarán parte de los archivos de salud mental de su hijo/a.

¿Cuáles son los riesgos y posibles incomodidades?

Usted o su hijo/a pueden cansarse de las preguntas de las evaluaciones, y algunas preguntas podrán preguntar sobre temas de las que quizás no querrán hablar. Usted y su hijo/a pueden decidir no responder cualquiera pregunta y pueden parar cualquiera evaluación a cualquier tiempo. También hay la posibilidad de que la confidencialidad de usted o su hijo/a sea violado; sin embargo, tomaremos grandes medidas para proteger la privacidad de usted y su hijo/a y, por lo tanto, minimizar este riesgo.

¿Cuáles son los posibles beneficios de participar en este estudio?

No se le puede prometer a usted y su hijo/a que habrá beneficios por participar en este estudio. Sin embargo, la terapia es diseñada para ayudar a los jóvenes y adolescentes con problemas relacionados a la ansiedad, la depresión, el trauma o la falta de conducta. También es posible que hablar con los investigadores durante las evaluaciones le ayude a usted o su hijo/a. Además, la información que se obtiene en este estudio puede ayudar a familias en el futuro a través de su contribución al mejoro de servicios de salud mental juveniles.

¿Qué pasa si decido participar, pero luego cambio de opinión?

Usted y su hijo/a pueden decidir parar el estudio en cualquier momento. Esta decisión no impactará a los servicios que ya usted o su hijo/a tenga el derecho a recibir. Si su hijo/a desea retirarse del estudio, puede discutir esta decisión con su hijo/a. Si siguen en desacuerdo, la participación de su familia en la investigación será descontinuada. Si deciden descontinuar su participación, es posible que le pidamos su permiso para incluir la data que se recolectado de usted y su hijo/a en nuestro estudio.

¿Puede mi hijo todavía recibir servicios de salud mental en nuestra clínica local comunitario de salud mental **si decidimos no participar en este estudio?**

Si, su hijo puede seguir recibiendo servicios de salud mental en su clínica local comunitario de salud mental si decide no participar en este estudio. Su decisión no cambiará el cuidado que recibe su hija/a ahora o que recibirá en el futuro. Formar parte de esta investigación es su decisión. Si usted y su hijo/a decide participar en este estudio, puede dejar o parar el estudio a cualquier momento. No habrá ningunas penalizaciones hacia usted o s u hijo/a, y el cuidado de salud mental de su hijo/a no será afectado. Si decide parar su participación en este estudio, por favor déjaselo saber a un miembro del equipo de investigación inmediatamente.

¿Hay recompensa por mi participación en este estudio?

Usted y su hijo/a serán recompensados por su participación en evaluaciones semanales y trimestrales (a los 0, 3 , 6, 9, 12 y 18 meces). La tabla de abajo resume la recompensa para cada evaluación.

Tipo de Evaluación	Tiempo dedicado por el/la Cuidador/a	Recompensa de Cuidador/a	Tiempo dedicado por el/la hijo/a	Recompensa de hijo/a
Evaluación Inicial	90 minutos	$30	60 minutos	$10
Evaluaciones Semanales	10 minutos	$5	10 minutos	$2.50
Evaluaciones Trimestrales	60 minutos	$30	30-60 minutos	$10
Evaluaciones a los 6 y 9 meses	60 minutos	$40	30-60 minutos	$15
Evaluaciones a los 12 meses	60 minutos	$50	30-60 minutos	$20
Evaluaciones a los 18 meses	60 minutos	$60	30-60 minutos	$25

¿Cuáles gastos tendré que cubrir yo si participo en esta investigación?

Usted pagaría por las sesiones de terapia en su clínica local de salud mental bajo las mismas circunstancias y costos para las familias que normalmente buscan servicios terapéuticos allí (por ejemplo, pueden ser reembolsado/a por Medicaid o su seguro privado). Nosotros recompensaremos a las clínicas por participar en el estudio para asegurar de que no pierdan ingresos o tiempo del personal (por ejemplo, en ser entrenados o participar en consultas) que socavaría sus servicios usuales hacia sus comunidades. Si su familia es asignada al nuevo tratamiento (Tratamiento #1), usted no incurrirá costos adicionales comparado al costo asociado con el acceso a los servicios terapéuticos usuales de su clínica.

Si formo parte de esta investigación, ¿Cómo será protegida mi privacidad? ¿Qué hacen con la información que obtienen?

Cada esfuerzo se hará para limitar el uso y divulgación de la información personal de usted y su hijo/a, incluyendo archivos del estudio y de salud mental, a personas que necesitan revisar esta información, incluyendo representantes del IRB. Solamente los investigadores tendrán acceso a la información de su familia—y solamente para los propósitos a las cuales usted y su hijo/a están de acuerdo. Usaremos números de identificación codificadas en todos archivos electrónicos y grabaciones audio o video del

estudio, cuales se mantendrán en computadoras seguras y protegidas con contraseña, plataformas y/o redes. Para permitirnos a contactarle/a a usted y a su hijo/a, mantendremos su información de contacto en un archivo digital protegida con contraseña y apartes de la data de la investigación. Cualquier otro archivo electrónico que tengan datos potencialmente identificativos serán guardados aparte también. La data de este estudio será una fuente valiosa de información sobre la terapia juvenil en clínica local comunitario de salud mental. Por lo tanto, mantendremos la data del estudio por tiempo indefinido, usándolo y compartiéndolo—de forma no identificable solamente—para propósitos de investigación. No compartiremos ninguna información que podrá identificar a usted o su hijo/a. El uso y compartimiento de la data del estudio será supervisado por un comité cuya función es asegurar que los derechos de usted y su hijo/a como participantes de la investigación son protegidos. Data grabada en audio o video se guardará hasta que se complete el estudio y toda la información del estudio haya sido completamente analizado. Después de eso, las grabaciones serán borrados permanentemente (a menos que usted nos da consentimiento explicito y aparte para que se guarden las grabaciones de audio o video para razones educativas o de entrenamiento).

¿Hay excepciones a la confidencialidad?

La confidencialidad no incluye información sobre posibles casos de abuso/la negligencia infantil o casos de riesgo considerable de daño a si mismo u otros. Si nuestro equipo de investigación recibe este tipo de información, es requerido por ley que tomemos las medidas necesarias para protegerle/la a usted, a su hijo/a o a otros de daño. Esto puede incluir que reportemos ciertos riesgos a los altos oficiales del equipo de investigación, autoridades apropiadas, el/la terapeuta de su hijo/a o a cualquiera persona quien puede estar en peligro.

¿Qué más debo saber sobre la confidencialidad?

Una descripción de este estudio se puede encontrar en http://www.ClinicalTrials.gov, según los requerimientos de la ley estadounidense. Esta página web no incluirá información que puede identificarle/la a usted o a su hijo/a. Como máximo, la página web incluirá un resumen de los resultados. Usted puede buscar esta página web en cualquier momento. Para ayudarnos en proteger la privacidad suya y de su hijo/a, esta investigación es cubierto por un Certificado de Confidencialidad del Instituto Nacional de Salud. Los investigadores pueden usar este Certificado para legalmente negarse a divulgar información que puede identificarle/la a usted o a su hijo/a en procedimientos federales, estatales, o local civil, criminal, administrativo, legislativo u otro tipo de procedimiento (por ejemplo, si hay una citación judicial). El Certificado de Confidencialidad no se utilizará para prevenir divulgación de cualquiera información que se haya recibido sobre un posible abuso infantil o riesgo de perjudicarse uno mismo o a otros a autoridades estatales o locales.

¿Qué más debo saber?

Esta investigación es financiada por el Instituto Nacional de la Salud. Dr. Weisz y Dr. Bearman, los investigadores principales de este estudio de investigación son profesores en la Universidad de Harvard y Universidad de Texas, respectivamente, que requiere que toda facultad divulga información financiera en las formas de consentimiento de los estudios de investigación. Dr. Weisz y Dr. Bearman, los autores y editores de múltiples manuales de tratamientos y libros sobre la terapia para jóvenes, adolescentes y familias, y son elegible para recibir regalías de los editores. Por lo tanto, ellos pueden recibir un poco de ingreso de los editores si los enfoques de tratamientos de las que ellos han escrito son exitosos y otra gente las usa. Por favor, pregunte cualquiera pregunta que usted o su hijo/a tenga sobre este tema.

Grabaciones para Propósitos Educativos y de Entrenamiento

Consentimiento para Tomar Parte en un Estudio de Investigación de los Humanos

Como se ha mencionada previamente, las sesiones de terapia de su hijo/a serán grabadas en audio o video. Estas grabaciones se usarán para propósitos de investigación solamente, y la identidad de su familia se mantendrá confidencial. Cuando el estudio ya se haya completado y toda la información de la investigación ha sido obtenido y analizado, estas grabaciones serán permanentemente borradas. Sin embargo, si nos permites a retener las grabaciones de audio o video después del estudio, solamente se usarán para propósitos educativos y de entrenamiento. La identidad de su familia se mantendrá confidencial. Su voluntad de permitirnos mantener estas grabaciones no afectará la habilidad de su familia de participar en este estudio ni tampoco afectará los servicios de salud mental que recibe su hijo/a. Por favor indica su decisión abajo:

- □ **Si, voluntariamente doy permiso** al equipo de investigación a retener y usar *grabaciones de video* de las sesiones de terapia de mi hijo/a para razones educativas o de entrenamiento tras la fecha de finalización del estudio.
- □ **Si, voluntariamente doy permiso** al equipo de investigación a retener y usar *grabaciones de audio* de las sesiones de terapia de mi hijo/a para razones educativas o de entrenamiento tras la fecha de finalización del estudio.
- □ **No, no doy permiso** al equipo de investigación a retener y usar *grabaciones de audio o video* de las sesiones de terapia de mi hijo/a para razones educativas o de entrenamiento tras la fecha de finalización del estudio.

Uso de los Archivos de la Clínica de Salud Mental para Propósitos de Investigación
Debido a los propósitos de este estudio, estamos solicitando acceso a los archivos que corresponden a su hijo/a de su clínica local comunitario de salud mental. Acceder a los archivos clínicos de su hijo/a **es requerido** para usted y su hijo/a participar en este estudio.

Si, voluntariamente doy permiso al equipo de investigación a usar y retener los archivos clínicos de mi hijo/a por propósitos de investigación.

No, no doy permiso al equipo de investigación a usar y retener los archivos clínicos de mi hijo/a por propósitos de investigación.

Uso de Información Obtenido Durante la Entrevista Calificativa Telefónica para Propósitos de Investigación

Usted compartió bastante información útil sobre su familia durante nuestra conversación inicial a través del teléfono (La Entrevista Calificativa Telefónica), como la edad de su hijo/a y como se han sentido recién. Necesitamos esta información para este proyecto y, en vez de pedirle que lo repita todo de nuevo, quisiéramos mantener esta información y combinarla con la información que compartirá durante su participación en este estudio.

- □ **Si, voluntariamente doy permiso** al equipo de investigación a usar y retener la información que compartí durante el proceso de la Entrevista Calificativa Telefónica para propósitos de investigación.
- □ **No, no doy permiso** al equipo de investigación a usar y retener la información que compartí durante el proceso de la Entrevista Calificativa Telefónica para propósitos de investigación.

Declaración de Consentimiento

Consentimiento para Tomar Parte en un Estudio de Investigación de los Humanos

He leído la información de esta forma de consentimiento incluyendo los riesgos y posibles beneficios. Todas mis preguntas sobre la investigación han sido respondidas a mi satisfacción. Entiendo que mi hijo/a y yo podemos retirar del estudio en cualquier momento sin penalidad o perdida de beneficios a las cuales ya estoy autorizado/a.

Firma

Su firma abajo indica que entiende lo que acaba de leer, que hemos respondido sus preguntas y que estás de acuerdo con participar y permitir a su hijo/a a participar en este proyecto de investigación.

____

Nombre de Participante

____ ____________________________________

Firma de Participante **Fecha**

____ ____________________________________

Firma de Persona obteniendo permiso **Fecha**

____

Nombre escrito de Persona obteniendo permiso

[*o*, en casos donde socios en la clínica están de acuerdo que no es posible obtener una firma digital]

Por seleccionar el sí abajo, indica que ha entendido lo que acaba de leer, que le respondimos todas sus preguntas y que está de acuerdo con su participación en esta investigación.

- ☐ Sí, estoy de acuerdo con mi participación en el estudio.
- ☐ No, no estoy de acuerdo con mi participación en el estudio.
