# Supplementary material for: Principle-Guided Psychotherapy for Children and Adolescents (FIRST): study protocol for a randomized controlled effectiveness trial in outpatient clinics
Source: Trials. 2023 Oct 21;24:682. doi: 10.1186/s13063-023-07717-y (PMC10589969; doi:10.1186/s13063-023-07717-y)
Supplement: Supplementary file 3 — Additional file 3. Assent to take part in a human research study. [file 13063_2023_7717_MOESM3_ESM.pdf]

Assent to Take Part in a Human Research Study

**Protocol Title:** Testing FIRST in Youth Outpatient Psychotherapy	
**Principal Investigator:** John R. Weisz, Ph.D.	
**Description of Participant Population:** Families (youths ages 7-15 and their caregivers) seeking community-based mental health services for anxiety, depression, trauma, or misconduct.	
**Version Date:** 8.27.21	

1. You and other children are being asked to take part in a research study at Harvard University. A research study is a way to learn more about something. We are trying to learn more about the best way to help kids deal with feeling sad, nervous, or angry. You are being asked to join this research study because you and your family are interested in getting treatment for some of these problems from a therapist in your community.

2. Whatever you say today or in the future will be private. No one will use your name to talk about anything that you say or do without your permission. But, if you tell us that someone is in danger—like that you have tried to hurt yourself or someone else; or, that someone has been hurting you—we will have to tell your caregiver (your parent or legal guardian) or someone else to keep you and others safe.

3. If you decide you want to join our study, we will begin by asking you some questions about your feelings. If you answer these questions today, you will receive a $10 electronic gift card.

4. As part of the project, you and your caregiver will start meeting with a therapist in your local community. These meetings will happen about once a week. Your therapist will make an audio- or video-recording of the meetings so we can know what happens.

5. During the project, we will get in touch with you to find out how you are doing and feeling. We will ask you to answer a few quick questions each week using an online survey. You will get a $10 electronic gift card for every 4 weekly surveys that you complete. Once every few months, we will call you on the phone to ask some more questions. You can earn between $10 and $25 in electronic gift cards for answering these questions.

6. You do not have to join this project. It is up to you. You can say okay now and change your mind later. All you have to do is tell us that you want to stop. No one will be mad at you if you don’t want to be in the study or if you change your mind later and stop. You can talk to your caregiver before you decide. If you don’t want to join or decide to stop but your caregiver wants you to join or keep going, we will respect your wish.

7. If you join the project, you might get tired of some of the questions or you might not want to answer some of them. You don’t have to answer any questions that you don’t want to, and you can stop whenever you like.

8. We do not know if being in this project will help you. What you learn in therapy might help you find ways to feel less sad, nervous, or angry. You may like talking to your therapist. You

might also like to know that what we learn from the project could help other kids and their parents feel better.

9. Drs. Weisz and Bearman are two of the people working on this project. They have written many books about how to help kids and their families when they are feeling sad, nervous, or angry. If this project helps some kids, then people might want to buy their books. Your name will not be used in any books about this project.

10. Before you say yes or no to being in this study, we will answer any questions you have. If you join the study, you can ask questions at any time. Just tell a member of the research team when you talk to them on the phone.

11. Your therapist will make audio- or video-recordings of what you talk about during therapy. These recordings are part of the study. If it's okay with you, we would like to keep these recordings for as long as we need them. We might use parts of the recordings to teach other people how to use some of the skills you learn in therapy. We will not use your name, and we'll keep any other information about you private. It is okay for you to say no. If you decide that you don't want us to keep the recordings after the study is over, no one will be mad. You can still be in the project if you say no. Is it okay for us to keep your recordings after the project is over?

☐ **Yes, it's okay** to keep and use my recordings after the project is done.
☐ **No, it's not okay** to keep and use my recordings after the project is done.

Let us know if you have any questions about this project!

If you sign your name below, it means that you understand what you just read, that we answered your questions, and that you agree to take part in this research study.

Child/Adolescent Assent

______________________________ ________________
Name of Participant Date

______________________________ ________________
Signature of Participant Date

______________________________ ________________
Name of Person Obtaining Assent Date

______________________________ ________________
Signature of Person Obtaining Assent Date

[*or*, in cases where clinic partners agree that it is not feasible to obtain a digital signature]

By selecting yes below, that means that you understand everything you just read, that all your questions were answered, and that you agree to participate in the research study.

☐ **Yes, I agree to particiate in the study.**

☐ **No, I do not agree to participate in the study.**
