# Supplementary material for: Principle-Guided Psychotherapy for Children and Adolescents (FIRST): study protocol for a randomized controlled effectiveness trial in outpatient clinics
Source: Trials. 2023 Oct 21;24:682. doi: 10.1186/s13063-023-07717-y (PMC10589969; doi:10.1186/s13063-023-07717-y)
Supplement: Supplementary file 4 — Additional file 4. Clinician consent to take part in a human research study. [file 13063_2023_7717_MOESM4_ESM.pdf]

Consent to Take Part in a Human Research Study

**Protocol Title:** Testing FIRST in Youth Outpatient Psychotherapy	
**Principal Investigator:** John R. Weisz, Ph.D.	
**Description of Study Population:** Families (youths ages 7-15 and their caregivers) seeking mental health services for anxiety, depression, trauma, or conduct problems.	
**Version Date:** 07-15-2021	

Key Information

You are invited to participate in a research study directed by Dr. John Weisz, a professor and clinical researcher at Harvard, and Dr. Sarah Kate Bearman, a professor and clinical researcher at the University of Texas at Austin. More detailed information is provided later in this form.

Why am I being invited to take part in a research study?

We have invited you to take part in this research study because you provide mental health services to children and families through a community mental health service organization with which we are partnering in order to test the effectiveness of a new treatment approach.

What should I know about a research study?

- A member of our research team will explain this research study to you.
- Whether or not you take part is voluntary. It is your choice whether to participate.
- You can choose not to take part in the study.
- You can agree to take part and later change your mind.
- If you choose not to participate or later decide to withdraw from the study, your decision will not be held against you. There will be no penalty or loss of benefits to which you are otherwise entitled.
- You can ask all the questions you want before you decide.

Why is this research being done?

The purpose of the study is to test the effectiveness of two different mental health treatment approaches for problems related to anxiety, depression, trauma, or misbehavior in children and adolescents. In **Treatment #1**, participating clinicians will be trained and receive consultation in the use of a new mental health treatment approach shown to be effective elsewhere and now being used in the community mental health organization where you work. In **Treatment #2**, therapists will rely on their previous training and experience. Some families in the research study will receive Treatment #1, and some families will receive Treatment #2. As a therapist, the treatment approach you use with participating families will be determined randomly (like the flip of a coin). Depending on this, you may receive training in Treatment #1 prior to the study and weekly case consultation during the study. All therapists in the Treatment #2 condition will be invited to be trained in Treatment #1 after treatment has been completed for all youths in the study. We will compensate you directly or via your organization for your study-related activities that are outside your normal work duties, at a rate similar to that paid by your employer and as approved by your employer. The study will help us to understand how helpful the new mental health treatment approach is.

How long will I take part in this research?

The duration of your study participation will vary, as the length of treatment for each child is variable. The study is expected to last for a total of five years, and you may choose to participate in one, some, or all of these years. By signing this form, you are giving us permission to contact you in the future.

What will my participation in the study involve?

Therapists who participate in the project will be randomly assigned (using procedures like a coin toss) to either **Treatment #1** (a new mental health treatment approach) or **Treatment #2** (the existing mental health services you typically provide at your clinic). If you are assigned to Treatment #1, you will receive training and weekly case consultation from Treatment #1 experts and then be asked to use this treatment with children and families participating in the study. The training will take place in segments over multiple days, lasting a total of 18 hours. Consultation will involve weekly group meetings (often via phone or video for about 1 hour per week) with Treatment #1 experts and other therapists. If you are assigned to Treatment #2, you will be asked to provide therapy as you ordinarily would in your clinic, using the approaches you think are most appropriate. At the end of the study, therapists in this condition will be offered the opportunity to receive training in Treatment #1. Audio- or video-recordings of therapy sessions are required for therapists in both treatment conditions. All families participating in the study will have agreed to have their therapy sessions recorded. The recordings are needed so that study coders can note the procedures used, for reports on study findings. The recordings will be retained until all study information has been collected and all data analyses are complete, and only retained in cases where you and a family has given us explicit permission to retain these recordings for educational and training purposes. Finally, you will be asked to complete a few survey measures. Compensation will be provided to you and/or your organization for measures you complete as well as for training, consultation, and other study activities that fall outside of your work requirements. More detailed information about the study procedures can be found under the *"What can I expect if I take part in this research*?" section.

Is there any way being in this study could be bad for me?

Audio- or video-recording therapy sessions and participation in weekly consultation meetings (for therapists assigned to Treatment #1) may result in some discomfort. If you experience such discomfort, your expert consultant or a research team member will gladly discuss this with you. More detailed information about the risks of this study can be found under the *"What are the risks and possible discomforts?"* section.

Will being in this study help me in any way?

We cannot promise any benefits to you from taking part in this research. The opportunity to learn new therapy procedures may benefit you in your work. Information from this study may also be helpful to therapists and families in the future by contributing to improved youth mental health services.

What happens if I do not want to be in this research?

Participation in this research study is completely voluntary. You can decide to participate or not to participate without any penalty whatsoever. If you decide to participate now, you may withdraw from the study at any time without penalty and without loss of benefits to which you are entitled.

Detailed Information

To follow, please find more detailed information about this study than already provided above.

About this consent form:
Please read this form carefully. It provides important information about participating in research. You have the right to take your time in making decisions about participating in this research. If you have any questions about the research or any portion of this form, you can ask us at any time. If you decide to participate in this research study, you will be asked to sign this form. A copy of the signed form will be provided to you for your record.

Who can I talk to?
If you have questions, concerns, or complaints, Dr. John Weisz, the principal investigator of this study, can be reached at Harvard University, 1030 William James Hall, 33 Kirkland Street, Cambridge, MA 02138 or by email at john_weisz@harvard.edu. This research study has been reviewed by the Committee on the Use of Human Subjects (CUHS) at Harvard University. If you wish to speak to a representative from the IRB, you may contact (617)-496-2847 or cuhs@harvard.edu for any of the following:

- If your questions, concerns, or complaints are not being answered by the research team.
- If you cannot reach the research team.
- If you want to talk to someone besides the research team.
- If you have questions about your rights as a research participant.
- If you want to get information or provide input about this research.

Participation is voluntary.
Participation in research is completely voluntary. It is your choice whether to participate or not to participate without any penalty whatsoever. If you decide to participate now, you may withdraw from the study at any time without penalty and without loss of benefits to which you are entitled. Refusal to participate or ending your participation will have no impact on your relationship with your clinic.

How many people will take part in this research?
Approximately 210 families and 40 therapists are expected to take part in this research study.

What can I expect if I take part in this research?
As a participant, you will be expected to complete the following. First, you will be invited to participate in the study at the mental health organization where you work. A member of the research team at Harvard will contact you to obtain your informed consent. If you consent to participate in the study, you will be randomly assigned to either Treatment #1 (i.e., a new mental health treatment approach) or Treatment #2 (i.e., the existing mental health services you typically provide at your clinic, which you deem to be most appropriate for youth and families participating in the study). If assigned to Treatment #1, you will receive training prior to treating study participants. This training will take place in multiple segments for a total of 18 hours. You will then be asked to use procedures from Treatment #1 with youth and families from your clinic participating in the study. You will also receive weekly group case consultation with study experts, for approximately 1 hour per week (including via phone or video) throughout the study. If you are assigned to Treatment #2, you will be asked to provide therapy as you ordinarily would, using the approaches you think are most appropriate with youth and families participating in the study. After treatment has been completed for youths in the study, you will be offered the opportunity to receive training in Treatment #1. Regardless of the condition to which you are assigned, you will be required to make audio- or video-recordings of therapy sessions with participating youths and families as part of

your participation. All participating families will have agreed to have their sessions recorded. The recordings are needed so that study coders can note the procedures used, for reports on study findings. The recordings will be retained until all study information has been collected and all data analyses are complete, and only retained in cases where you and a family has given us explicit permission to retain these recordings for educational and training purposes Finally, you will be asked to complete study measures, including questions about therapy practices and your satisfaction with the treatment procedures you used in the study.

What are the risks and possible discomforts?
Audio- or video-recording therapy sessions and participation in weekly consultation meetings (for therapists assigned to Treatment #1) may result in some discomfort. There is also the possibility that your confidentiality could be breached; however, we will take great efforts to protect your privacy and thus to minimize this risk.

Are there any benefits from being in this research study?
We cannot promise any benefits to you from taking part in this research. However, the opportunity to learn new therapy procedures may benefit you in your work. Information from this study may also be helpful to therapists, families, and clinics in the future by contributing to improved youth mental health services.

What happens if I say yes, but I change my mind later?
You can leave the research study at any time without penalty. This decision will not impact your relationship with the clinic where you work. If you decide to discontinue participation, we may ask for your permission to include data previously collected from you in our study.

Will I be compensated for participating in this research?
Compensation will be provided to you and/or your organization for measures you complete as well as for training, consultation, and other study activities that fall outside of your work requirements.

What will I have to pay for if I participate in this research?
It will not cost you anything to participate in this research.

If I take part in this research, how will my privacy be protected? What happens to the information you collect?
Every effort will be made to limit the use and disclosure of your personal information to people who have a need to review this information, including representatives from the IRB. Only researchers will have access to your information—and only for the purposes to which you agree. We will use coded identification numbers on all research study forms, files, and audio- and video-recordings, which, when applicable, will be kept in a locked and secure location. Computerized and digital data will be stored on secured computers and networks using confidential usernames and passwords. To permit us to contact you, we will keep your contact information in a locked filing cabinet and password-protected computer file, separate from research data. Any other documents that have potentially identifying data will be stored separately as well. Data from this study will be valuable sources of information about therapy for youth in community-based mental health clinics. Therefore, we will keep study data indefinitely, using and sharing it—in de-identified form only—for research purposes. We will not share any information that could identify you. The use and sharing of study data will be overseen by a committee whose role is to ensure that your rights as research participants are protected. Audio- and video-recording data will be kept until the study is complete and all study information has

been fully analyzed. After that, the recordings will be permanently deleted (unless you give us separate/explicit consent for recordings to be kept for educational/training purposes).

Are there any exceptions to confidentiality?
Confidentiality does not extend to information about possible child abuse or significant risk of harm to self or others. If we are given such information, we are required by law to take necessary actions in order to protect participants or others for harm. This may include reporting risks to senior members of the research team, appropriate authorities, or any person who might be in danger.

What else should I know about confidentiality?
To help us protect your privacy, this research is covered by a Certificate of Confidentiality from the National Institutes of Health. The researchers can use this Certificate to legally refuse to disclose information that may identify you in any federal, state, or local civil, criminal, administrative, legislative, or other proceedings (for example, if there is a court subpoena). The Certificate of Confidentiality will not be used to prevent disclosure to state or local authorities of any information receive about possible child abuse or risk for harm to self or others.

What else do I need to know?
This research is being funded by the National Institutes of Health. Dr. Weisz and Dr. Bearman, the principal investigators of this research study, are professors at Harvard University and the University of Texas, respectively, and both universities require their faculty to disclose financial information in research study consent forms. Dr. Weisz is an author and editor of multiple treatment manuals and books on therapy for children, adolescents, and families, and he and Dr. Bearman are co-authors of the treatment program that will be used in this study. Both are eligible to receive royalties from their publishers. Thus, both can receive some income from publishers if the treatment approaches they have written about are successful and other people use them. Please ask any questions that you might have about this.

Recordings for Educational and Training Purposes
As mentioned previously, therapy sessions will be audio- or video-recorded. These digital recordings will be used for research purposes only, and your identity will be kept confidential. Once the study is complete and all study information has been collected and analyzed, these recordings will be permanently deleted. However, if you and a participating family provides us with permission to retain these recordings after the study, they may be only used for educational and training purposes. Your identity will remain confidential. Your willingness to allow us to keep these recordings will not affect your ability to participate in this study nor your relationship with the clinic in which you work.

☐ **Yes, I willingly give permission** for the research team to retain and use *audio-recordings* of therapy sessions for educational or training purposes beyond the completion of this study.

☐ **Yes, I willingly give permission** for the research team to retain and use *video-recordings* of therapy sessions for educational or training purposes beyond the completion of this study.

☐ **No, I do not give permission** for the research team to retain and use audio- or video-recordings of therapy sessions for educational or training purposes beyond the completion of this study.

Statement of Consent

Consent to Take Part in a Human Research Study

I have read the information in this consent form including risks and possible benefits. All my questions about the research have been answered to my satisfaction. I understand that I am free to withdraw at any time without penalty or loss of benefits to which I am otherwise entitled.

Please indicate whether you would like to participate in this study by checking the appropriate box below.

☐ **Yes, I would like to participate in this study.**
☐ **No, I would not like to participate in this study.**
